# ‘Ship-in-a-Bottle’ Integration of pH-Sensitive 3D Proteinaceous Meshes into Microfluidic Channels

**DOI:** 10.3390/nano15020104

**Published:** 2025-01-10

**Authors:** Daniela Serien, Koji Sugioka, Aiko Narazaki

**Affiliations:** 1 Innovative Laser Processing Group, Research Institute for Advanced Electronics and Photonics, National Institute of Advanced Industrial Science and Technology (AIST), Tsukuba 305-8568, Ibaraki, Japan; 2 Advanced Laser Processing Research Team, RIKEN Center for Advanced Photonics, Wako 351-0198, Saitama, Japan

**Keywords:** femtosecond laser direct writing, 3D printing, microfluidic integration, pH-actuation

## Abstract

Microfluidic sensors incorporated onto chips allow sensor miniaturization and high-throughput analyses for point-of-care or non-clinical analytical tools. Three-dimensional (3D) printing based on femtosecond laser direct writing (fs-LDW) is useful for creating 3D microstructures with high spatial resolution because the structures are printed in 3D space along a designated laser light path. High-performance biochips can be fabricated using the ‘ship-in-a-bottle’ integration technique, in which functional microcomponents or biomimetic structures are embedded inside closed microchannels using fs-LDW. Solutions containing protein biomacromolecules as a precursor can be used to fabricate microstructures that retain their native protein functions. Here, we demonstrate the ship-in-a-bottle integration of pure 3D proteinaceous microstructures that exhibit pH sensitivity. We fabricated proteinaceous mesh structures with gap sizes of 10 and 5 μm. The sizes of these gaps changed when exposed to physiological buffers ranging from pH of 4 to 10. The size of the gaps in the mesh can be shrunk and expanded repeatedly by changing the pH of the surrounding buffer. Fs-LDW enables the construction of microscopic proteinaceous meshes that exhibit dynamic functions such as pH sensing and might find applications for filtering particles in microfluidic channels.

## 1. Introduction

The integration of diverse functionalities onto microfluidic chips is key to realizing micro-total analysis systems such as organ-on-a-chip and lab-on-a-chip constructs [1,2,3]. On-chip microfluidic systems incorporate multi-faceted technologies that mimic specific physiological environments and functionalities. For example, an organ-on-a-chip mimics the environment and functionality of a human organ [1]. As a step in this direction, three-dimensional (3D) microfluidic cell culture systems serve as non-clinical human tissue models [2]. Microfluidic systems are miniaturized systems that consume little reagent, support rapid chemical reactions, and are amenable to running parallel experiments. These advantages allow high-throughput testing and support the customization required for point-of-care applications [3].

The selection of materials for large-scale manufacturing is a bottleneck in applying microfluidic systems to industrial applications [3]. Microfluidic system injection molding is the most common fabrication method used, and 3D printing approaches are also widely utilized because they are accessible, more cost-effective than injection molding, and can be used with biocompatible materials. Currently, 3D printing is mostly used in microfluidic research for prototyping and preliminary studies [3]. Increased access to high-performance laser equipment will particularly aid the adoption of optical 3D printing and accelerate the use of this technique on an industrial scale [4].

Femtosecond laser direct writing (fs-LDW) is a versatile 3D optical printing approach with high spatial resolution and excellent 3D fabrication capability [5,6]. Two-photon polymerization processes using ultrafast lasers are often used to confine the processed regions in a 3D manner and reduce the effective spot size in transparent media due to multiphoton absorption [7,8]. Fs-LDW using a transparent precursor material that fills a glass microfluidic device can provide embedded functional micro-components or biomimetic structures within closed glass microchannels (‘ship-in-a-bottle’ integration), creating high-performance 3D biochips [9].

Proteins dissolved in aqueous solvents are one example of a transparent medium for fs-LDW. Proper selection of the energy and photon intensity during optical printing can provide proteinaceous micro- and nanostructures that retain the native protein function. Applications such as micro-optics, pH actuation, cell culture scaffolds and enzymatic nanoreactors have been previously reported [10]. Methacrylated protein was recently used to tune the Young’s modulus of a printed material by adjusting the photoresist composition in scaffolds for cell culture [11]. Intricate 3D protein microstructures have been reported for precursor from unmodified protein and photosensitizer [12,13,14] and pure unmodified protein [15]. Although the fabrication mechanism using the pure protein precursor is not fully understood, we recently demonstrated that chemical crosslinking is involved [15] similar to the case of using photosensitizers [13,16], and more recently we identified the amino acids that might aid the fabrication process, by comparing fabrication results obtained using commercially available homopeptides [17]. Biocompatible applications using integrated microfluidic chips might particularly benefit from the use of pure protein precursors to prevent undesired leaching of photoactivators after fabrication, since photoactivators might reduce cell viability or cause other side effects [18].

The integration of proteinaceous microstructures with photoactivators into microfluidic channels was previously demonstrated. An enzyme was incorporated as individual lines in a glass capillary and exhibited enzymatic activity. The activity of this miniaturized channel could be measured [19]. Similarly, the integration of trypsin columns into a polydimethylsiloxane microfluidic channel confirmed its compatibility with classic microfluidic channels [20]. Using antibody protein, the capture of living cells in a biochip was demonstrated [21]. We demonstrated that refractive index matching enabled fs-LDW of 3D microstructures in closed microchannels via the ship-in-a-bottle integration technique [22].

The integration of proteinaceous microstructures exhibiting pH sensitivity [23] into microfluidic chips could have interesting applications. pH-sensitive proteinaceous devices have found utility in tunable micro-optics [24], artificial musculoskeletal systems [25] and shape-shifting structures [26]. However, these studies were conducted using structures created in a dish or on a glass substrate, which obscured the spatial and temporal resolution of the pH measurements. The integration of microfluidic channels might help to control volume changes and buffer flow.

**Figure 1:**
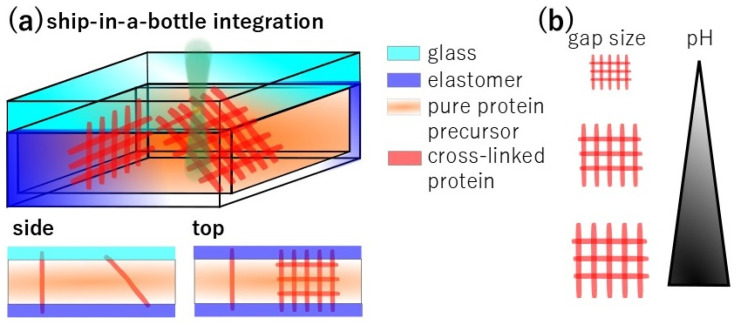
Schematic drawing showing integration of pure 3D proteinaceous mesh into glass-elastomer microchannels. (**a**) A thin cover glass is bonded to the elastomer microchannel to enclose the microchannel. The closed microchannel is filled with a high concentration of protein precursor solution. Fs-LDW is used to fabricate proteinaceous meshes in the channels which are anchored at the top and bottom of the channel. (**b**) In the pH range 4–10 under our fabrication conditions, the gap size of meshes correlates with the pH of the applied external pH buffer solution.

In this paper, we demonstrate the integration of photoactivator-free pH-sensitive pure 3D proteinaceous meshes in a closed microfluidic channel. As shown in Figure 1a, the closed microchannel is filled with a pure, high-concentration protein precursor solution. Fs-LDW is used to fabricate proteinaceous meshes in the channels which are anchored to the top and bottom of the channel. The meshes allow accurate measurement of pH-dependent changes in the size of the mesh. The conditions used for fs-LDW fabrication are important for pH sensitivity. Using the chosen fabrication and precursor conditions, the relationship between pH and gap size allows manipulation of the gap size to adjust pH sensing, as indicated in Figure 1b. The ship-in-a-bottle approach using pure protein allows pH control and should thus facilitate the development of active protein-based microdevices with practical applications in biomedicine.

## 2. Materials and Methods

### 2.1. Protein Precursor Preparation and Free-Standing Fabrication

Bovine serum albumin (BSA) (pH 5.2 fraction, 017-21273 (Lot no. WTP3913), Fujifilm, Tokyo, Japan) was purchased as a lyophilized powder. The protein powder was weighed and dissolved in purified water or a 50% (*w*/*w*) glycerol−water solvent to 400 mg/mL (~6 mM) BSA.

We evaluated the pH -actuation of protein meshes unconstrained by a microchannel by fabricating meshes anchored onto cover glass without restricting the mesh itself. The precursor was drop-cast using 3 μL drops onto the same type of thin cover glass as used previously for channel bonding (120–170 μm-thick cover-glass; Matsunami, Osaka, Japan), then the cover slip was inverted to form a hanging drop to mimic the in-channel fabrication path from the fs laser objective lens, through air, to the thin cover glass and into the protein precursor.

### 2.2. Setup

**Figure 2:**
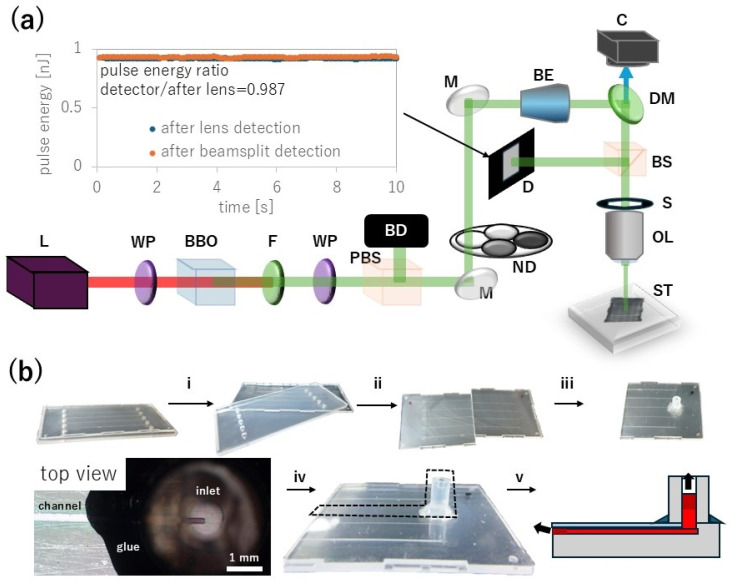
Fabrication setup and microfluidic chip preparation. (**a**) The 1040-nm laser (L) output with a repetition rate of 100 kHz and a pulse width of about 350 fs is converted to 520 nm green laser light via lambda-half zero order waveplate (WP), beta barium borate crystal (BBO) and the remaining NIR is filtered by a 520 nm bandpass filter (F). PC-controlled power attenuation, which is achieved via another waveplate (WP) and a polarizing beam splitter (PBS) that leads excess energy into a beam dump (BD), mirrors (M), and neutral density (ND) filters set the pulse energy. A 2x beam expander (BE) overfills the back-end aperture of the N.A. = 0.45 objective lens (OL) with a working distance (WD) of 13.8 mm. Through a 50:50 beam splitter cube (BS), the average power is monitored in situ with a microscope slide power sensor (D). The inset shows the measured pulse energy converted from power measurements by taking into account a transmittance of 0.987 for both split paths. A dichroic mirror (DM) and camera (C) allow in situ observation. Mechanical shutter (S) and mechanical stage (ST) are PC-controlled. (**b**) (i) A microfluidic chip is split into elastomer and hard-plastic top cover. (ii) The elastomer is cut. (iii) After cleaning the surface with ethanol, an O_2_ plasma is applied for 60 s to facilitate bonding and increase channel hydrophilicity. Then, the O_2_ plasma-treated 120 μm-to 170 μm-thin glass is quickly and firmly attached to the channel to be bonded, as well as a 5 mm-long silicon tube aligned to the inlet. (iv) After a further 30 s of O_2_ plasma, the channel is filled with protein precursor, as indicated with dashed outline. (v) Evaporation from the inlet and outlet causes flow inside the microchannel. Therefore, we store the channels for at least 1 day until the evaporation solidifies the precursor at inlet and outlet, causing the in-channel liquid precursor to become stationary.

An fs laser (Spirit 1040-16-HE-SHG, Spectra-Physics, Milpitas, CA, USA) operated at a wavelength of 1040 nm, a pulse width of about 350 fs, and a repetition rate of 100 kHz was used as a light source. The near infrared (NIR) Gaussian beam was converted to 520 nm green laser light and the remaining NIR was filtered and removed by a bandpass filter, as shown in Figure 2a. Proteins generally only strongly absorb light at wavelengths shorter than ultraviolet (UV) and thus the two-photon absorption process at the focus of the green laser pulse can excite proteins and form proteinaceous fine structures [27]. Fs-LDW requires precise control of the laser pulse energy, and thus the pulse energy was monitored in real time through a 50:50 beam splitter cube with a PD300-MS microscope slide power sensor (Ophir, Jerusalem, Israel).

### 2.3. Microchannel Preparation

A microfluidic chip was prepared using a commercial product, a Microfluidic Kit Natural Flow Chip Type III (Richell, Toyama City, Japan), as shown in Figure 2b. The product comes with a 1 mm-thick plastic cover. The working distance of the lens can accommodate this thickness but the plastic cover is slightly cloudy white, reducing the focus of the laser on the microfluidic channels. Therefore, we separated the plastic cover into two components. With scissors, we cut the channel in half to increase test samples, then we wiped the elastomer components with ethanol to remove adhesive residue, and subjected them to O_2_ plasma treatment for 60 s in an O_2_ plasma device (SP-0400, Strex, Osaka, Japan). Then, the simultaneously O_2_ plasma-treated 120–170 μm-thick cover-glass (Matsunami, Osaka, Japan) was immediately firmly bonded to the microchannel. Further, a 5 mm-long silicon tube (part of the kit) with an inner diameter of 1 mm was bonded on top of the channel inlet.

After confirming proper alignment between tube and channel inlet, we used 5 min super glue for glass surfaces and enhanced the tube to channel bond. Then, we treated the channel with 30 s O_2_ plasma to prepare for filling the microfluidic chip with precursor. Less than 10 μL of 400 mg/mL protein precursor was pipetted into the inlet per chip. The precursor was sufficiently viscous to provide the desired fs-LDW results but not so viscous as to prevent its flow into the channel due to capillary action, and thus the channel was filled without the use of an external pump.

For several hours after the channel was filled, fs-LDW fabrication cannot be executed reliably because the evaporation from the inlet and outlet and capillary flow along the walls of the channel cause precursor solution to move inside the channel. Especially at slow fabrication speed such as our designated fabrication speed of 5 μm/s across our chosen channel dimensions of 100 × 50 μm^2^, already fabricated parts of the mesh will dislodge or detach before the whole mesh can be finished. Therefore, we stored the protein-solution-filled channels for at least 1 day at room temperature and approximately 40% relative humidity, so that evaporation significantly reduced upon the formation of solidified protein plugs at the inlet and outlet while retaining protein precursor liquid in the inner channel.

Due to reduced evaporation, precursor flow inside the channel halts nearly completely when a few millimeters away from the inlet or outlet. We found that after 1 day, this condition of minimal flow is reached and fs-LDW fabrication can be executed with high reliability. The necessary storage time is likely dependent on precursor composition, channel dimensions and inlet/outlet geometry, with room temperature and relative humidity playing a secondary role. After fs-LDW, the solidified protein plugs were gently rehydrated with buffer solution and all of the excess protein precursor rinsed by repeatedly adding buffer to the inlet.

### 2.4. Buffer Exchange

We used the following standard pH solutions: Phosphate buffer pH 6, carbonate pH standard solution (pH 10), pH 4.01, pH 9.18 and pH 6.86 buffers from Fujifilm Wako Chemical (Osaka, Japan) and pH standard solutions (technical type) of pH 5 and pH 8 from Hanna Instruments (Woonsocket, RI, USA). Fluorescein sodium salt (F6377-1006, Sigma-Aldrich, Tokyo, Japan) fluorescent dye was used to determine that the speed of gravitation flow was approximately 30 μm/s in the center of the channel (Figure S1 in the Supplementary Information).

### 2.5. Data Analysis

The data are presented as the mean and standard deviation for n samples (number of physical samples). Mesh gap sizes were observed using a bright-field microscope (BX53, Evident, Tokyo, Japan) and analyzed with ImageJ (version1.54j, National Institutes of Health, Bethesda, MD, USA) by manual read-out. We attempted to automate the analysis (see Supplementary Information Table S1), but unfortunately it was very difficult due to contrast differences between images and due to meshes bending in and out of the image focus.

Free-standing microstructures were imaged with a 20× magnification lens immersed in the respective buffer. Scale bar distortion due to liquid immersion was evaluated using fixed dimensions embedded into the cover glass surface and the obtained corrective factors were applied to the image measurements. The 20× images without lens immersion had a scale of 155 pixels to 50 μm, whereas the buffer-immersed images had a scale of 155 pixels to 41.67 μm.

The laser scanning fluorescence images were reconstructed into 3D format using an FV3000 laser scanning microscope (Evident, Tokyo, Japan) and the integrated SV31-SW software v.2.6.1. Images were obtained with an NA = 0.85 40× lens using 405 and 488 nm excitation lasers to excite the autofluorescence of cross-linked proteinaceous materials.

## 3. Results

The pH actuation effect of 10-μm and 5-μm gaps in the fabricated proteinaceous meshes was evaluated. We first evaluated the pH sensitivity of meshes not integrated in channels and then that of integrated proteinaceous microstructures.

### 3.1. Free-Standing Mesh Fabrication

#### 3.1.1. Design and Free-Form Fabrication

**Figure 3:**
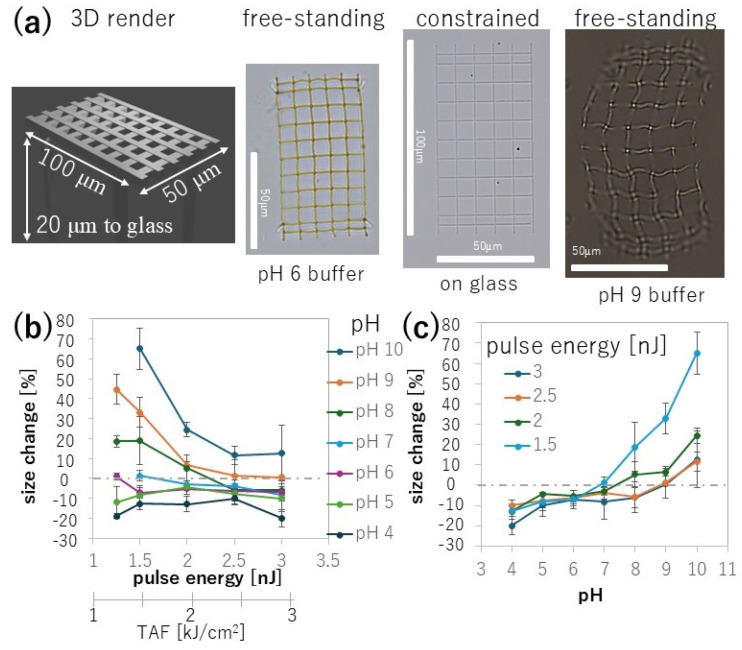
Design of 3D free-standing mesh and constrained on-glass control. (**a**) A 3D rendered image made from Blender (v 2.92.0, Blender Foundation, Amsterdam, The Netherlands) represents the dimensions of the mesh. The 3D render is to remind that fs-LDW forms volume elements and even though the top down view looks 2D, each line element is actually 3D of micron-to-submicron scale. Three microscopic images are shown. A 100 × 50 μm^2^ area is fabricated with either 10 or 5 μm-wide gaps between the mesh lines. Immersion in pH 6 (left) and pH 9 (right) buffer shows that the meshes are free-standing. The mesh gap size changes from its original size, depending on the external pH. The original design is shown as a control attached to glass (middle). Attachment to the glass surface constrains the structure and prevents changes in the gap size. Scale bars represent 50 μm. (**b**) Gap sizes were evaluated as the percent change as a function of the pulse energy used for fabrication. At the given stage scanning speed, the fabrication window is approximately 1.25 to 3 nJ. On a secondary *x*-axis in (**b**), we converted the pulse energy to total accumulated fluence (TAF). (**c**) Recontextualized data of (**b**) are plotted as the dependence of gap size on the pH value for pulse energies between 1.5 and 3 nJ. The gap size monotonically increased with increasing pH for 1.5 nJ, but for 2, 2.5 nJ and 3 nJ are non-monotonic with fluctuations.

After creating free-standing structures on a glass surface, we can observe the meshes detached from the glass and study for the first time the unrestrained deformation of the meshes resulting from pH changes. The concept behind our approach is shown in Figure 3a. After forming the free-standing structure, the microstructure was rinsed to remove excess precursor present in the buffer. The anchoring feet were fabricated using a 1 nJ laser and a stage scanning speed of 5 μm/s, extending 5 μm further into the glass to ensure adequate anchoring despite surface irregularities or errors in identifying the surface on the order of several micrometers.

The integrity of the end of each line comprising the mesh was affected by the acceleration and deceleration of the mechanical stage during fs-LDW fabrication. Even at low laser energies, the total fluence delivered to the mesh line was much higher when the stage slowed to a halt or just began its acceleration. As we describe below, the amount of energy delivered to the mesh line affected the final shape of the mesh. We therefore studied relatively large meshes, with an area of 100 × 50 μm^2^ so that we could ignore such effects at the edge of the mesh, where the scanning speed decreases, and evaluate only the center of the meshes for changes in gap size.

The free-standing mesh was evaluated by top-down microscopic observation to obtain the gap size and overall dimensions. Because the mesh was free-standing, 3D bending might occur. In the top-down observation, we observed such 3D deformation when the image was out of focus. Example images of free-standing meshes anchored through feet on glass show significant changes in gap size in pH 6 buffer and pH 9 buffer compared to that of the original fabricated structure, as shown in Figure 3a.

#### 3.1.2. pH-Sensitivity

We used free-standing meshes fabricated on glass substrates to quantitatively evaluate the percentage change in the gap size at various pH values. Two complete data sets are shown in the Supplementary Materials in Tables S2 and S3. Figure 3b,c summarize the percentage change, ranging from −20% to +65%, for meshes in pH 4 and pH 10 buffer. There was no discernible difference between 5 and 10 μm-sized gaps.

The fabrication window ends at about 1.25 nJ. Structures fabricated at energies below this were visible due to chemical reactions but were insufficiently cross-linked to maintain their structure upon release of the precursor. The fabrication window then extends to just below 3 nJ, above which the mesh disconnected at several points. Such disconnection might be caused by microbubble generation due to laser-induced heating and breakdown of the solvent [28]. We used the total accumulated fluence (TAF) to characterize the fabrication window by fluence and pulse number, as these are both important parameters in fs-LDW (see equations in Supplementary Materials).

Figure 3c is a replot of Figure 3b as a function of pH value. Our determined fabrication window and the pH range tested provided a nonlinear, monotonic increase relationship. A monotonic relationship is important to uniquely relate gap size to pH for controlling the size of the gap and for pH sensing. Fabrication at the lowest pulse energy for reliable fabrication results 1.5 nJ appears suitable for high-sensitivity sensing due to the larger change in gap size per pH unit. Pulse energies of 2 and 2.5 nJ provide a moderate increase in gap size between well separated pH values such as pH 4 and pH 10, suitable for controlling the gap size while ensuring mechanically sturdy meshes due to decreased deformation, however they did not yield a monotonic relationship and fluctuations rather than continuous steps render pH 5 and pH 6 indistinguishable. Pulse energy of 3 nJ provides a comparable result to pulse energies of 2–2.5 nJ, but due to its higher energy delivery, the fabrication process is more prone to microbubble occurrences. A microbubble occurs due to local breakdown of the solvent, coinciding with higher temperatures in the range of 200 degree Celsius [28] and gas expansion. The rapid expansion disturbs the fabrication of a mesh locally and distorts dimensions such that evaluation might become challenging. Therefore, microbubbles are undesirable in fs-LDW with protein.

### 3.2. Ship-in-a-Bottle Integration Inside Channel

We integrated proteinaceous meshes into glass-elastomer microchannels by filling the channel with 400 mg/mL BSA precursor. The precursor was dropped onto an indented inlet, then spread by capillary flow. In-channel liquid precursor was stationary after evaporation solidified the precursor at the inlet and outlet for at least 1 day, see Figure 2b.

#### 3.2.1. 3D Fabrication

The microfluidic channels filled with the precursor were transferred into our fabrication device with the bonded cover glass facing up towards the laser focus. In-situ camera imaging allowed us to identify the microchannel walls. The laser focus at 2 nJ was moved at 5 μm/s diagonally in the channel several times to determine the channel ceiling and bottom coordinates in the *Z* axis. Due to the higher refractive index of the precursor and the glass, light propagation slightly elongated the location of the laser focus, and the true distance moved by the laser focus from the ceiling to the bottom of the 50-μm tall microchannel was about 65 μm. The fabrication coordinates were thus extended by several micrometers in both directions from the determined coordinates to ensure that the fabricated structures were anchored to the ceiling and bottom of the channel.

**Figure 4:**
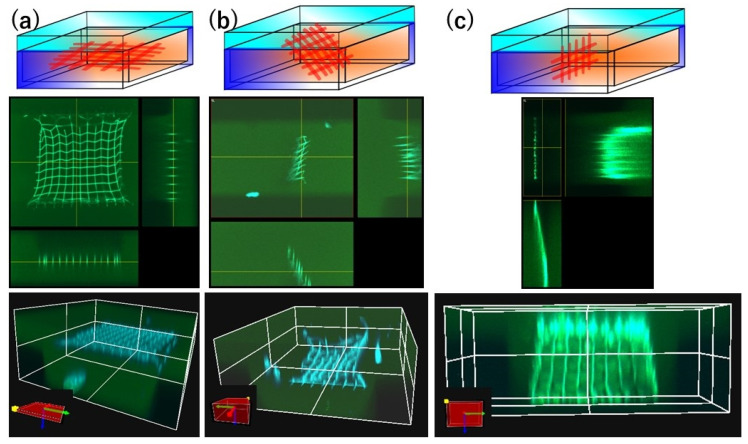
(**a**) Fluorescence laser scanning visualization of 3D proteinaceous meshes in a channel upon 405 and 488 nm excitation. (Top) Schematic representations, (middle) orthogonal views, and (bottom) 3D reconstructions are shown for (**a**) a horizontal design anchored to the channel side walls and fabricated with pulse energy of 2 nJ, (**b**) a diagonal design fabricated with pulse energy of 1.5 nJ and (**c**) a vertical design fabricated with pulse energy of 2 nJ for horizontal and 1.4 nJ for vertical lines. The diagonal and vertical meshes are anchored on the channel ceiling and bottom. All designs have a gap size of 10 μm and were fabricated with 400 mg/mL BSA embedded in 100 μm-wide microchannels with a 100 × 50 μm^2^ cross-section, using a laser energy of 1.4–2 nJ at a scanning speed of 5 μm/s. After removal of the precursor by rinsing with pH 6 buffer, a pH 6 buffer containing fluorescein sodium salt was capillary flowed into the channel to allow green fluorescence detection of the channel and blue fluorescence detection of the fabricated structures. The elastomer is not fluorescent and therefore shows as dark areas.

Figure 4 shows that we can anchor meshes in a horizontal, diagonal or vertical plane. We used the fluorescent dye fluorescein sodium salt to observe the dimensions of the channel by its green fluorescence. The uncross-linked precursor was completely removed from the entire channel by immersing the chips in a dish with several milliliters of buffer solution for at least one night.

The buffer had a pH of 6 as the gap size was essentially constant regardless of the pulse energy used for fabrication, as seen in Figure 3b; since pH 5 is the isometric pH of BSA and pH 7 is neutral, they would also work well. After removing the precursor by rinsing with buffer, buffer containing fluorescein sodium salt was introduced into the channel to label the created structures, enabling 3D imaging. The chips can be stored in either precursor or buffer to maintain the proteinaceous meshes.

#### 3.2.2. In-Channel pH Sensing

**Figure 5:**
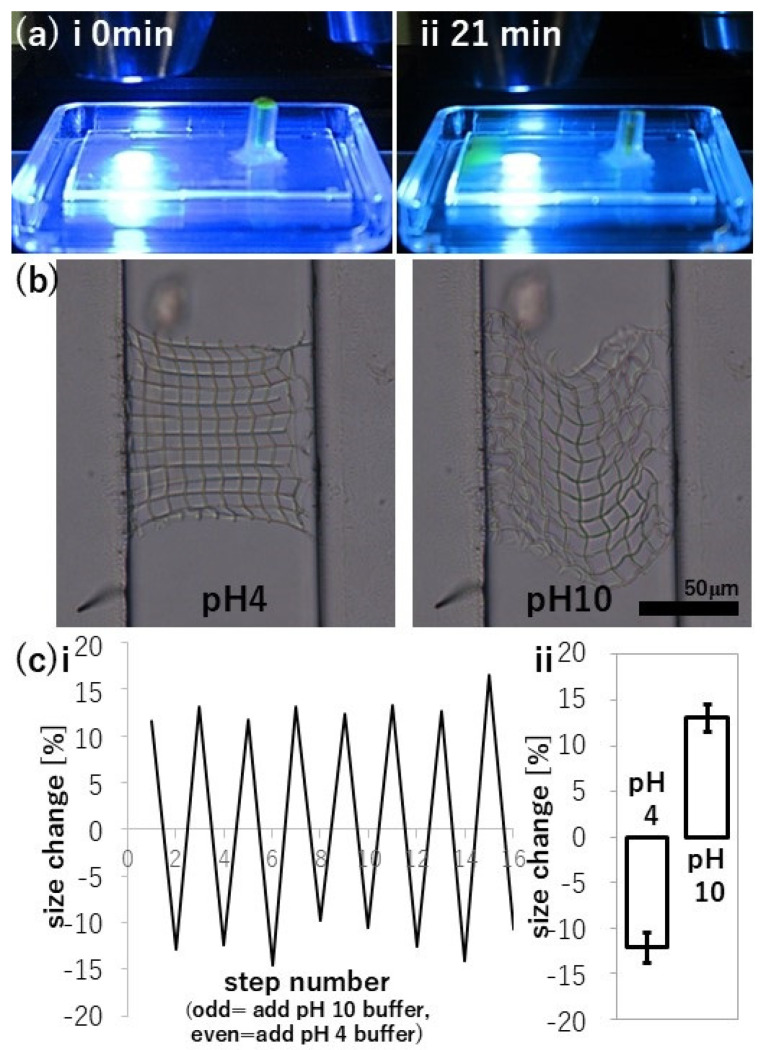
Setup, gap size and pH-actuation of in-channel meshes. (**a**) Filling the inlet with fluorescein sodium salt and pH 6 buffer to trace liquid movement. The gravitation flow resulting from a height difference of 5 mm was determined to be about 30 μm/s. To observe the microstructures with 20× lens, 12 mm or more distance to the inlet is required, resulting in 10–20 min long experiments for buffer exchange. The timing of refilling the inlet is also delicate, waiting too long and accidentally including air inclusion risks terminating the flow. Rough handling of the inlet can cause leakage which also terminates the experiment. (**b**) Deformation of a mesh fabricated using a laser energy of 2 nJ and a scanning speed of 5 μm/s in pH 4 and pH 10 buffer. (**c**) Change in gap size by repeatedly changing the pH of buffer solution after 10 buffer exchanges. (**i**) The pH 10 buffer is introduced at the odd-numbered step and the pH 4 buffer is introduced at the even-numbered step. (**ii**) In pH 4 buffer, the gap size was smaller by −12 ± 2% compared to the original size. In pH 10 buffer, the gap size increased by +13 ± 2%.

The precursor was removed from the fabricated meshes by immersion overnight in buffer solution, then excess buffer in the inlet was replaced by pipetting various buffers to a height of approximately 5 mm to allow consistent gravitational flow. Using fluorescein sodium salt, we visualized the flow in the device in Figure 5a. It takes circa 20 min for the buffer to flow and reach the sample site, which is located about 12–15 mm from the inlet to leave enough physical space for the 20× lens to image the sample. Every consecutive step of buffer exchange contains a high risk to accidentally end the gravitational flow because either air inclusions might cause the capillary flow to be disrupted or damages to the tube inlet might cause leakage at the base of the tube. The chosen glass-compatible glue was too stiff for long-term usage. Because of these difficulties, we commonly executed 3–5 steps of pH alterations. In principle, pH-response of proteinaceous structures is well demonstrated up to 200 cycles [25]; thus, we consider only the limiting factors here to be the robustness of the microfluidic channel and the ability to control and exchange liquid flow.

Because our device is driven by gravitational flow, buffer must be refilled with a pipette tip at the inlet or replaced with the next buffer to the same height. We chose this approach by gravitational flow despite the waiting time to avoid potentially damaging the mesh by manual pipetting and applying high uneven pressures.

We quantified the gap sizes by evaluating pH-actuation using horizontal and diagonal meshes, as these are easier to analyze by top-down microscopy compared to vertical meshes. Figure 5b shows the results for horizontal mesh fabricated at 2 nJ with 25 buffer exchanges (complete data set is shown in Supplementary Materials Table S4). After the first 10 buffer exchanges, mesh responsiveness became consistent (see Supplementary Materials Figure S2). Exposing the mesh to pH 4 buffer reduced the average gap size by −12 ± 2% from the 10-μm-sized design, while exposure to pH 10 buffer increased the average gap size by +13 ± 2%. These values roughly match the results shown in Figure 3b for the free-standing structure measurements. Figure 5c shows the reproducible changes in gap size for a few repetitions.

We noticed that complete mesh deformation takes about three times longer when changing from pH 4 to pH 10, in which deformation occurs in pH 10 buffer, than changing from pH 10 to pH 4, in which deformation occurs in pH 4 buffer. Due to the slow gravitational flow setup and frequent leakage at the inlet, it is hard to control the flow and volume in the microfluidic channel. Therefore, the quantitative values might vary but qualitatively it was reproducible. We found in the best case in which washing stabilized the performance that it took an average of 3.6 s for deformation in pH 4, while 11.1 s at pH 10 (see Supplementary Materials Figure S2). Due to changes in osmotic pressure, formation of the protein hydration shell and different diffusion times for the buffer ions into the proteinaceous mesh might be dependent on the initial buffer, resulting in these different response times.

## 4. Discussion

Pure proteinaceous meshes were integrated into a microchannel using the fs-LDW approach and the effect of buffer pH on the mesh gap size was evaluated. Within our fabrication window and the pH range tested, the mesh gap size increased mostly monotonically with increasing pH. Monitoring changes in the gap size enabled real-time sensing of the pH of the liquid buffer flowing in the microfluidic channel. The ability to control the gap size of a protein mesh by changing the pH of the surrounding buffer might allow cells and particles to be filtered using microfluidic devices.

These characteristics of pure 3D proteinaceous microstructures are distinctly different from those of microstructures reported previously. Specifically, two extreme pH values were previously required to activate switching states [25,26]—either pH 5 and pH 13 [25] or pH 5 and pH 11 [26]—or monotonic regimes were not identified by lack of selection of fabrication conditions [23]. Our findings of increasing swelling with higher pH are matching the reports for BSA pH responsiveness reported in [23] and [25,26], where our specific superiority is due to the selected fabrication conditions that utilize a narrow pH range to produce a monotonic relationship between the change in the gap size and the pH value.

In our pH exchange experiment, we utilize microchannel flow to precisely deliver the buffer to the mesh using a simple setup with gravitation and capillary flow. Deformation of the microstructure begins almost instantaneously upon changing the pH of the environment. More accurate timing to compensate for a possible sub-second delay would require the use of microfluidic pumps, but this would be challenging because we removed the plastic cover and replaced it with bonded glass, complicating the attachment of microfluidic tubing to reliably sustain pressure controlled by a microfluidic pump.

The literature demonstrates that pH-actuation of proteinaceous microstructures is likely due to charged surface residues affecting the expansion or contraction of the proteinaceous mesh [23,25]. More specifically, we suggest that surface charges and charged residues might affect the protein hydration shell (PHS). In contrast to photopolymer molecules, protein molecules are surrounded by a hydration shell comprising static and dynamical layers of water molecules. Such hydration shells are controlled by the surface properties of the protein and are also strongly influenced by the size and geometric shape of the protein [29].

All previous reports of proteinaceous microstructures consider the relationship between some fabrication conditions and microstructure swelling and shrinkage. Notably, Lee et al. investigated the effect of fabrication conditions on stiffness in pH response [26]. Some reports used hatching layer distance [26] or step length [25], which required maintaining the other parameters such as beam spot size. Total accumulated fluence might be a great candidate to compare results among different laboratories and laser fabrication setups. Our results are in agreement with [26] that less densely cross-linked networks swell and shrink more and are less mechanically robust.

A slower response time was observed when pH 10 buffer was used in comparison to when pH 4 was applied to the mesh. This can be explained by the response of the PHS to osmotic pressure, electrostatic repulsion, and molecule packing density. When the mesh is initially exposed to a lower pH environment, such as pH 4, the mesh experiences a lower osmotic pressure compared to that in a higher pH environment such as pH 10 [29]. Similarly, in a lower pH environment, amino acid residues are protonated and electrostatic repulsion is weaker than in a higher pH environment with deprotonated amino acid residues. Lastly, when pH 4 is used as the beginning state, the molecular network is condensed, slowing the diffusion of buffer ions, even though the diameter of pH 10 ions is smaller than that of lower pH ions.

Our time observations differed from those recently published elsewhere that measured protein shrinkage and swelling through the displacement of attached polymer components [25]. This group worked with pH 1 and pH 13 solutions, which are quite extreme pH values that denature BSA, given that BSA denatures at pH 3 and pH 11 [30], which might contribute to the differences from our findings since denaturation would change the tertiary structure and thus the surface charges. They reported several response times depending on their specific device design, each with very high repeatability. In our experiment, about 1–2 s of standard deviation existed even when using the same device, and leakage and a flow variation within the microfluidic channel that might further cause a negative effect with each new buffer exchange. Ref. [25] reported that the response time of pH 5 was very similar to that of pH 13 for two different designs, one design with 1.4–1.5 s and the other with circa 1 s each. For a third design, they reported about 3 s for pH 5 while less than 2 s for pH 13. In contrast, we observed the 3 times longer response time for pH 10. The trend of longer time for pH 10 was observed throughout different experiments. As proposed in the previous paragraph, the relationship between pH 4 and pH 10 can be explained via PHS. The other groups reported different results due to use of extreme pH, or because pH 5 is close to the isoelectric point for BSA which creates a different dynamic of surface charges in response to pH changes. Our results might differ from previous works due to microfluidic flow, interplay with surface charges of the elastomer or the use of standard solutions of buffers.

The highly pH-sensitive meshes with a low crosslink density used in the current study allow us to determine the pH from the gap size, because the gap size changes greatly in response to changes in the pH values in the environment. The error of those deformations resulting in gap size changes is approximately 3%, allowing pH determinations with at least integer accuracy. Improved gap size analysis methods to compensate for mesh irregularities and out-of-plane bending might provide finer resolution.

We demonstrated ship-in-a-bottle integration in 100 × 50 μm^2^ channels in a 3D manner. Furthermore, we showed that the relationship between gap size and pH matched the results obtained using free-standing structures. Slight differences observed between the two approaches appear to be due to the microfluidic channel slightly restricting mesh gap expansion at higher pH values, leading to a reduction to 13% from the expected 24% at pH 10. Channel restrictions can further increase the risk of entanglement during swelling and straining of the meshes, which might break the anchorage in channel walls during shrinkage. In Table S5, we show examples of detached and well-attached meshes. When properly attached, the mesh can withstand repeated use and rough treatment. It is not fully clear what improves anchorage, but O_2_ plasma treatment and embedded fabrication into the channel elastomer seem to have contributed to the attachment.

Our results demonstrate an approach to 3D fabrication to anchor proteinaceous meshes into microfluidic channels, supporting increased fabrication versatility compared to previous reports [19,20,21,22]. Our findings also show the potential applicability of integrated proteinaceous 3D structures for creating cell culture microenvironments to support the controlled growth of cell cultures or tissues on-chip. The scalability to commercial or industrial needs is not yet achieved, but with higher power lasers, it will become possible to compensate for the slow fabrication speeds of 5 μm/s to increase throughput by a parallel processing scheme using multi-spots.

Meshes for use as filtration layers with pH-sensitive gap sizes should probably be generated using a 2 nJ laser power setting rather than 1.5 nJ. Control of the gap size by the external pH is possible without compromising the robustness of the mesh required for filtration applications. The gap size can be changed in the range from −12 to +24 percent of the original size. An average gap of 10 μm encompasses the range from 8.8 to 12.4 μm. Choosing a smaller gap, such as 5 μm, could allow filtering of 4.4 to 6.2 μm-sized particles. However, a pH sensing application benefits from fabrication at 1.5 nJ because the gap size change in response to individual pH values is wider and the monotonic relationship is more pronounced, which increases the sensitivity. A greater change in response might be easier to trace by microscopic observation, and the resolution of the image is less affected.

This study was conducted entirely using BSA only. BSA can be considered the standard protein because it is prominently featured nearly always for laser 3D printing of protein [10]. The reason why BSA is so popular is that it is commercially available at an economic price, easily cross-links due to beneficial amino acids, and is quite easy to handle because of very basic storing and safety measures. In the comprehensive study of chemical responsiveness of 3D printed proteinaceous microstructures [23], it was demonstrated that three different protein types (BSA, avidin and lysozyme) had different pH responsiveness profiles (e.g., at specific pH values, BSA microstructures swell, while avidin microstructures shrink). Other protein types likely have other pH-dependencies, and as long as one can identify a monotonic relationship between the size change and pH, the concept of this work is transferable.

## 5. Conclusions

We demonstrated the 3D integration of pure proteinaceous microstructures into microfluidic channels and then applied the fabricated chips to study the pH responsiveness of the fabricated meshes. Our findings revealed unconventional monotonic relationships between the external pH and proteinaceous structure shrinkage or swelling. This relationship can be utilized to control the mesh gap size via pH change or to determine the in-channel pH via observation of the mesh gap size changes. The mesh gap size changes were reproducible over many pH change cycles. The response time of mesh deformation at pH 10 was 3 times longer than that at pH 4. This difference can be understood from differences in the PHS under these two pH conditions. Further improvements in mesh and microfluidic channel anchoring method, an automated microfluidic pump system including air-free liquid exchange ability, and strategies to avoid mesh entanglement will be important for future challenges. Ship-in-a-bottle integration for microfluidic applications may find applications in biomedical and microfluidic systems.

## Data Availability

A minimal data set is provided with the Supporting Information. The raw data supporting the conclusions of this article will be made available by the authors upon reasonable request, without undue reservation.

## Supplementary Materials

The following supporting information can be downloaded at: https://www.mdpi.com/article/10.3390/nano15020104/s1, Figure S1: Flow profile. (a) fluorescence image of flow, (b) binary image isolating the profile from (a), (c) joint image, (d) matching red-dashed u(y) to the flow profile; Figure S2: Response time. (a,b) Data from table S5 is shown for (a) size change and (b) response time. (a) In the first couple of steps size change in pH 10 buffer was not stable yet. (b) The black line shows time to maximal size change. The red line shows time to initial response. (c–i) Considering the first 10 steps as washing steps, the remaining 15 steps are shown. (ii) The averages of these 15 steps are shown. Average response times are 3.6 ± 1.3 s for a pH 10 to pH 4 change and 11.1 ± 1.6 s for a pH 4 to pH 10 change; Table S1: Automation attempt for image analysis; Table S2: Data set for pH 5 buffer for Figure 3; Table S3: Data set for pH 8 buffer for Figure 3; Table S4: Data for Figure 5; Table S5: Images of meshes before and after use.
