# Supplementary material for: ‘Ship-in-a-Bottle’ Integration of pH-Sensitive 3D Proteinaceous Meshes into Microfluidic Channels
Source: Nanomaterials (Basel). 2025 Jan 10;15(2):104. doi: 10.3390/nano15020104 (PMC11767245; doi:10.3390/nano15020104)

Supplementary Information

'Ship-in-a-Bottle' Integration of pH-Sensitive 3D Proteinaceous Meshes into Microfluidic Channels

Table of Content

1. Flow velocity estimate 2

2. Common equations 2

3. Examplary data sets for Figure 3 3

3.1 incomplete automation attempt 3

3.2 pH 5 data set 5

3.3 pH 8 data set 7

4. Data sets for Figure 5 8

4.1 2nJ horizontal mesh, 25 steps 8

4.2 response time 12

4.3 anchorage 13

1. Flow velocity estimate

We estimated speed from fluorescein sodium salt flow profile in the microfluidic channel with the equation:

$$u(y) = -\frac{1}{2\mu}\left(\frac{dP}{dx}\right)(h^2 - 4y^2),$$

where *u(y)* is velocity at the spatial coordinate *y*, which is perpendicular to the flow direction, μ is viscosity (estimated as 0.001 Pa s for water), *dP/dx* is the pressure gradient matched until the profile fit the observation (approximately -500 Pa/m) and *h* the channel width. The channel width is 100 μm, but the initial observation had still viscous precursor covering the walls with an effective channel width of approximately 50 μm, see Figure A1. Initial flow is estimated to be 60 μm/s, which decreases to 30 μm/s when the channel is properly rinsed. With distances of 12-15 mm between observation site and inlet, real time measurements took more than 10 minutes and match these estimates.

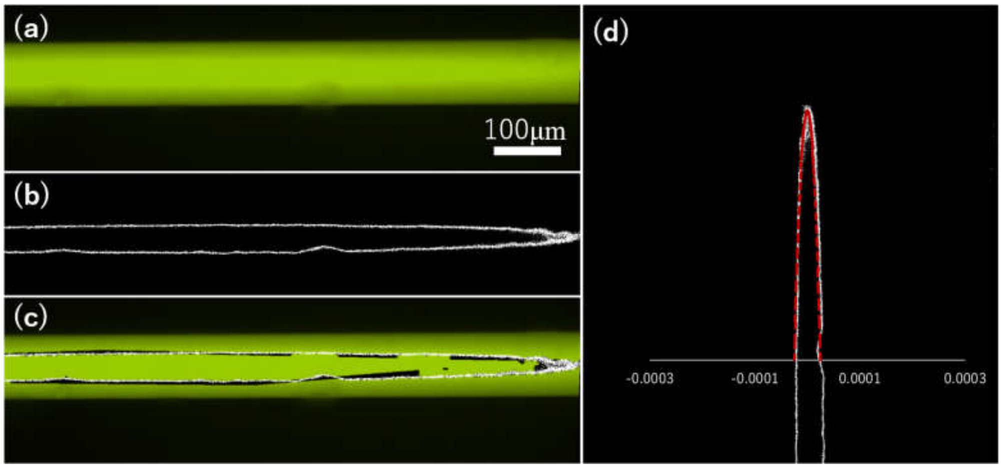

Figure S1: Flow profile. a) fluorescence image of flow, b) binary image isolating the profile from a), c) joint image, d) matching red-dashed u(y) to the flow profile.

2. Common equations

1) Beam radius:

$$\omega_0 = \frac{0.61 M^2 \lambda}{N.A.},$$

where M^2 is the beam quality, λ the center wavelength and N.A. numerical aperture.

2) **Fluence** Φ:

$$\Phi = E_{Pulse}/A_{focal\,spot},$$

where E_{Pulse} is the laser pulse energy [J] and $A_{focal\,spot}$ is the effective focal spot area [cm^2].

3) **Effective number of pulses, N_{eff}:**

$$N_{eff} = \sqrt[2]{\frac{\pi}{2}}\frac{\omega_0 f}{v},$$

where $\sqrt[2]{\frac{\pi}{2}}$ is the integration result for Gaussian beam geometric overlap, ω_0 is the beam radius [cm], f is the repetition rate [Hz], and v is the scanning velocity [cm/s].

4) **Total accumulated fluence TAF:**

$$\text{TAF} = N_{eff}\Phi$$

3. Examplary data sets for Figure 3

3.1 incomplete automation attempt

To convert mesh images into the information of mesh gap, we used ImageJ on microscopic images:

Convert 8bit and select a threshold to create a binary in which the mesh is represented in white (start with black, then invert). Process→Binary→Skeletonize. Crop and Analyze→Skeleton (2D/3D) with pruning of shortest branches, elimination prune ends, all results and output. The result ideally is 4 path lengths that represent each side length.

Reasons why this automation attempt was not further pursued:

- Contrast on the mesh can lead to shadows that interfere with a clean binary generation.
- Pruning does not always work. Preventing automated side length read-out.
- Side lengths are not recognized as one branch, then side length becomes fractured.
- Mesh knots might not be recognized as branch ends.
- Automation will overlook samples that bend in-or out of the image focal plane.

Table S1: Automation attempt for image analysis

Ideal case:
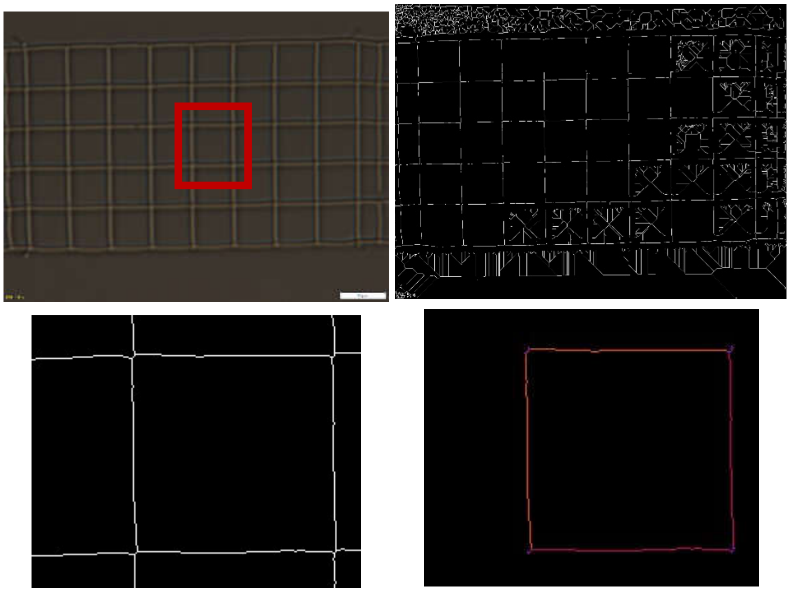
Common issue case: nodes are not correct, fractured side length and pruning was ineffective.
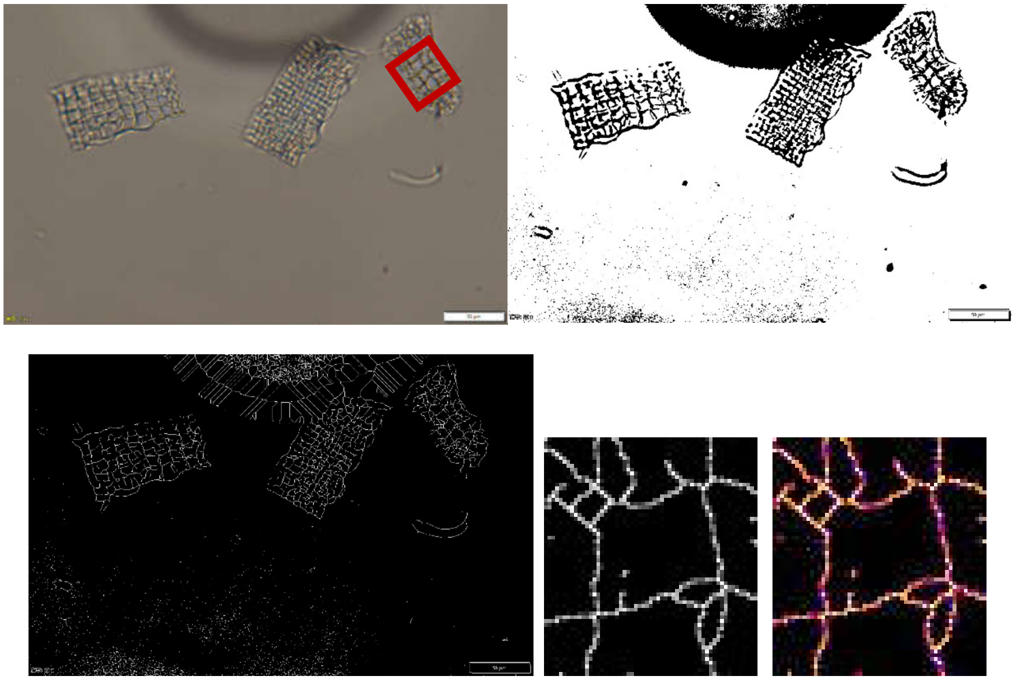

3.2 pH 5 data set

Table S2: Data set for pH 5 buffer for Figure 3

<table>
<tr><td colspan="2">Before (in precursor of 400mg/mL BSA)</td></tr>
<tr><td>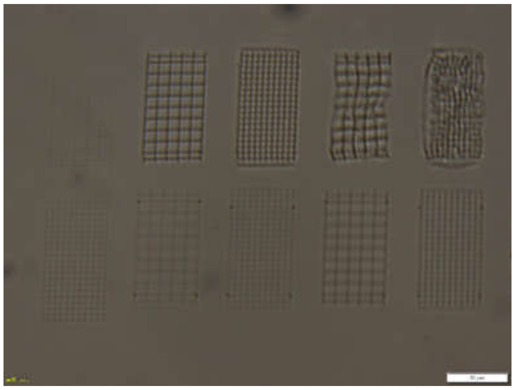</td><td>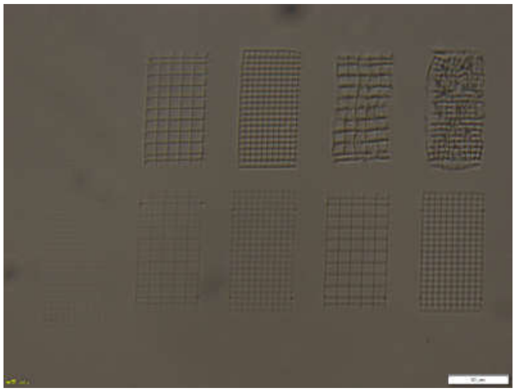</td></tr>
<tr><td>in focus : on glass surface,
upper row, very left: 10 µm, 2nJ, 5um/s.
lower row, very left: 5 µm, 2nJ, 5um/s.</td><td>in focus : free-standing meshes
upper row: 2nJ (10&5µm), 2.5nJ (10&5µm)
lower row: 1.25nJ (10&5µm), 1.5nJ (10&5µm)
3nJ set out of the image.</td></tr>
<tr><td colspan="2">Video 188 (real time, with 20x lens is immersed in buffer)
The 20x images without lens immersion had a scale of 155 pixels to 50 µm, whereas the buffer-immersed images had a scale of 155 pixels to 41.67 µm.</td></tr>
<tr><td colspan="2">After (20x lens immersed in pH 5) – different focal planes.</td></tr>
<tr><td>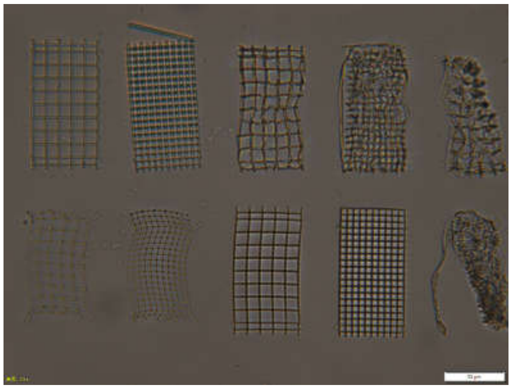</td><td>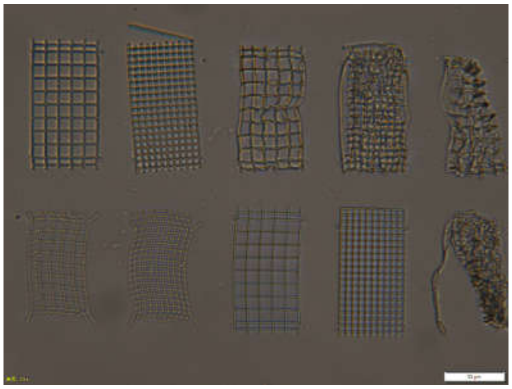</td></tr>
</table>

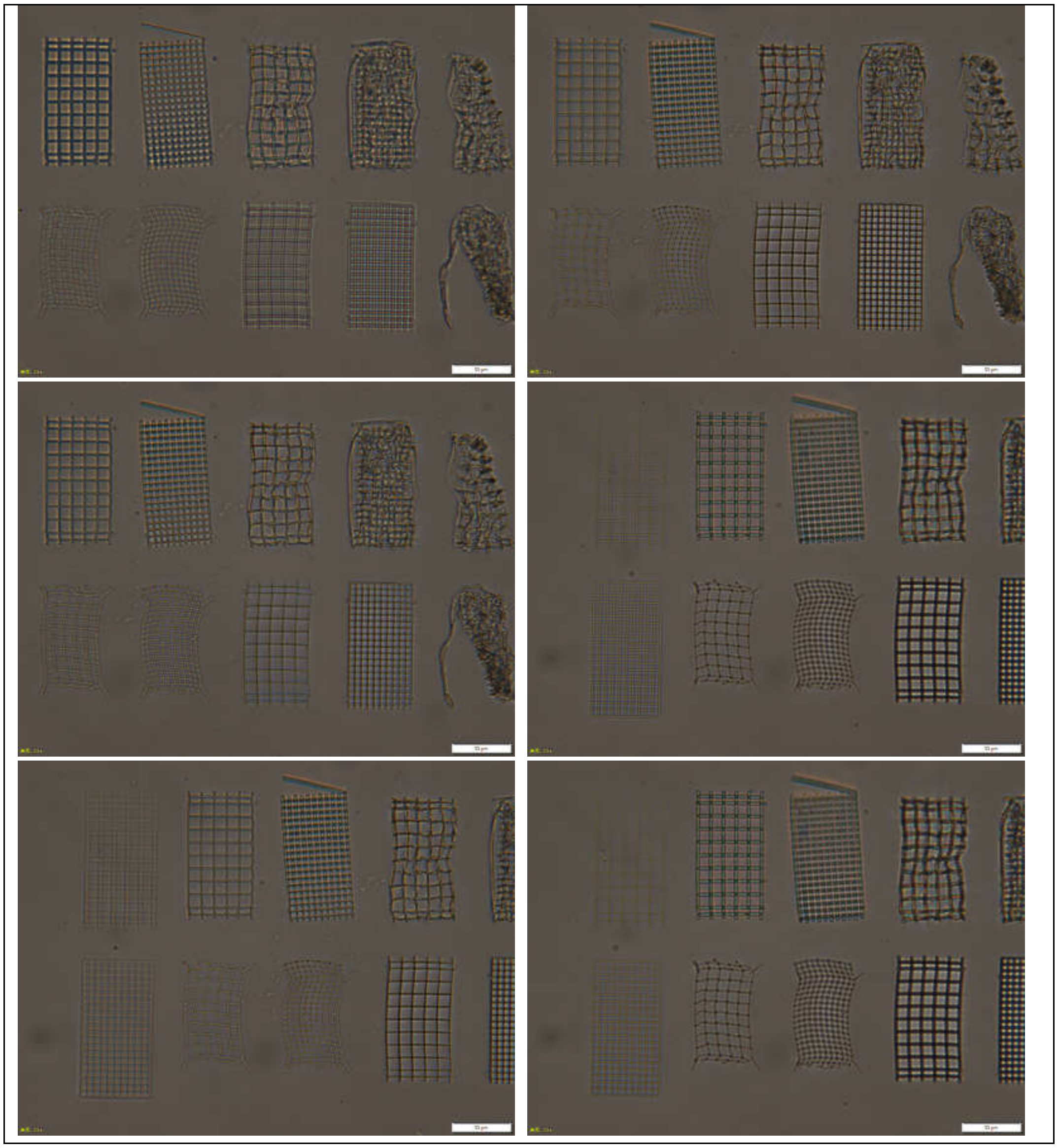

3.3 pH 8 data set

Table S3: Data set for pH 8 buffer for Figure 3

Before (in precursor of 400mg/mL BSA, imaged with a wetted lens due to previous experiment)
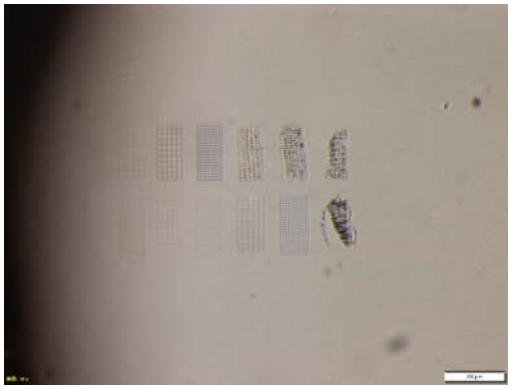 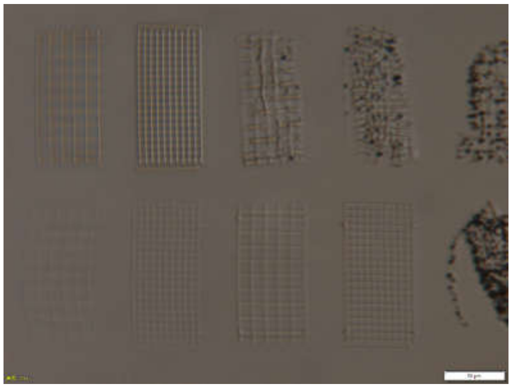
10x view , 20x view<br>upper row (from left): on glass (2nJ, 10µm), 2nJ (10&5µm), 2.5nJ (10&5µm), 3nJ (10µm)<br>lower row (from left): on glass (2nJ, 5µm), 1.25nJ (10&5µm), 1.5nJ (10&5µm), 3nJ (5µm)
Video 193 (real time, with 20x lens is immersed in buffer)<br>The 20x images without lens immersion had a scale of 155 pixels to 50 µm, whereas the buffer-immersed images had a scale of 155 pixels to 41.67 µm.
After (20x lens immersed in pH 8) – different focal planes.
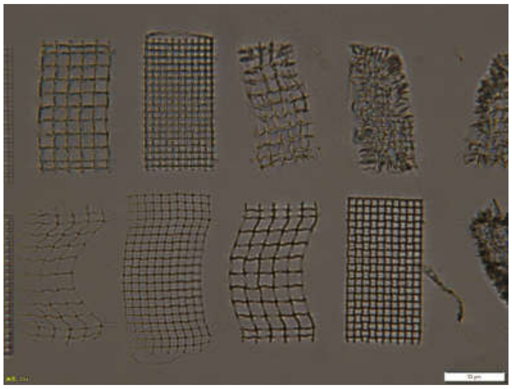 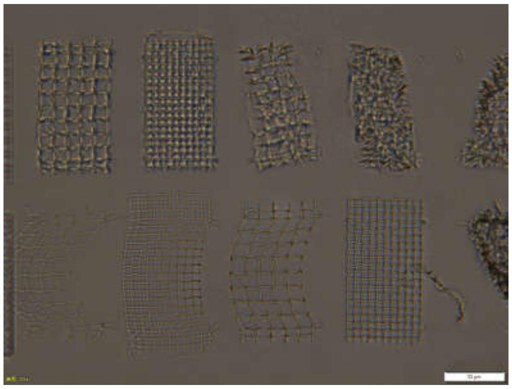

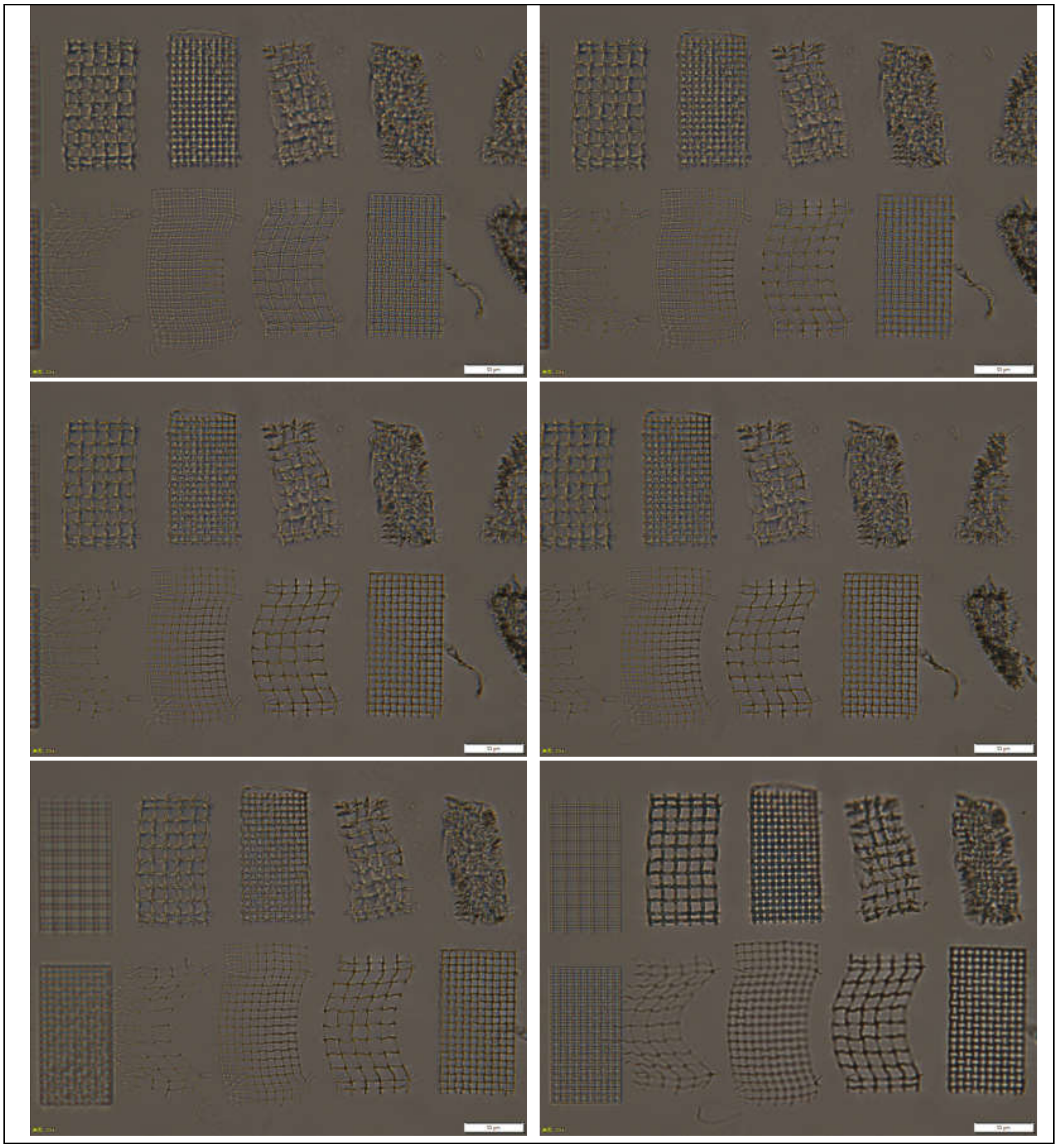

4. Data sets for Figure 5

4.1 2nJ horizontal mesh, 25 steps

Images are screenshots from videos, the timing was determined manually by carefully sliding through the frames to identify the timing of initial response and maximal expansion. The scale was determined to be 76 pixels to 50 micrometers with still images as well as 100μm width of the channel.

Table S4: Data for Figure 5

						**images**	**size change (over 10μm) [%]**	
before					**stored in pH 6**	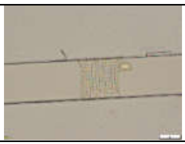	**-10.2475**	**transition**
								**count as wash**
after157				**pH 10 and pH 4 step mixed up**		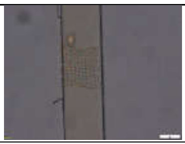	**-7.3025**	**1**
movie	from pH	to pH	seconds to **maximal form** from beginning of movie	seconds from **first! visual response**	**response time (s)**			
158	N.A.	4	N.A.	4.66	**N.A.**	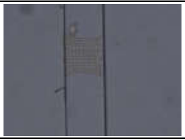	**-10.8275**	**2**
159	4	10	40.84	4.58	**36.26**	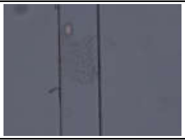	**5.965**	**3**
160	10	4	11.63	5.14	**6.49**	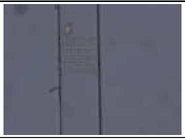	**-12.5425**	**4**
161	4	10	32.88	5.66	**27.22**	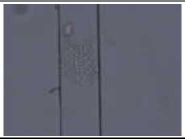	**9.215**	**5**
162	10	4	13.34	4.91	**8.43**	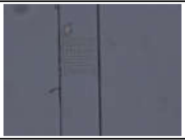	**-10.935**	**6**

163	4	10	20.97	5.68	**15.29**	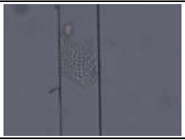	**12.4025**	**7**
164	10	4	8.46	3.52	**4.94**	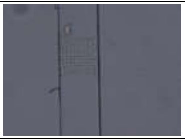	**-10.3375**	**8**
165	4	10	20.31	4.6	**15.71**	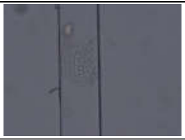	**13.33**	**9**
166	10	4	12.9	4.05	**8.85**	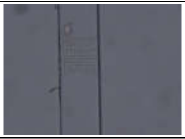	**-11.9175**	**10**
								**Count as measure**
167	4	10	17.06	3.71	**13.35**	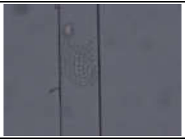	**11.5425**	**1**
168	10	4	8.27	3.93	**4.34**	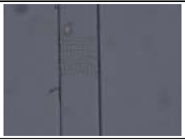	**-12.9225**	**2**
169	4	10	18.15	5.16	**12.99**	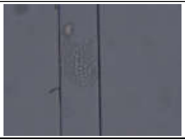	**13.0525**	**3**
170	10	4	12.02	5.6	**6.42**	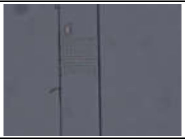	**-12.345**	**4**
171	4	10	16.58	5.11	**11.47**	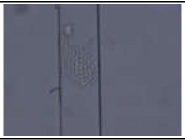	**11.645**	**5**
172	10	4	9.13	5.96	**3.17**	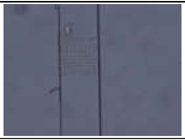	**-14.61**	**6**
173	4	10	16.64	4.74	**11.9**	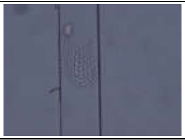	**13.1775**	**7**

174	10	4	8.82	6.2	**2.62**	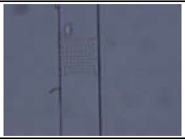	**-9.805**	**8**
175	4	10	17.52	7.39	**10.13**	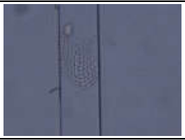	**12.265**	**9**
176	10	4	9.27	6.03	**3.24**	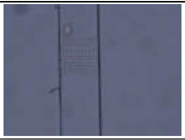	**-10.4875**	**10**
177	4	10	12.93	3.41	**9.52**	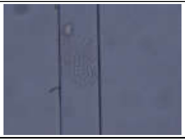	**13.2525**	**11**
178	10	4	7.89	5.54	**2.35**	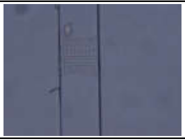	**-12.485**	**12**
179	4	10	15.11	5.21	**9.9**	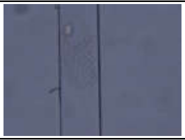	**12.69333333**	**13**
180	10	4	9.67	6.03	**3.64**	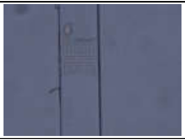	**-14.08**	**14**
181	4	10	13.44	3.97	**9.47**	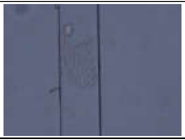	**16.46666667**	**15**
182	10	4	8.33	5.43	**2.9**	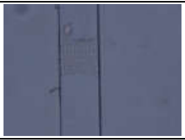	**-10.6375**	**16**
183	4	6				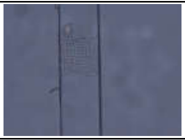	**-8.7925**	**transition**

4.2 response time

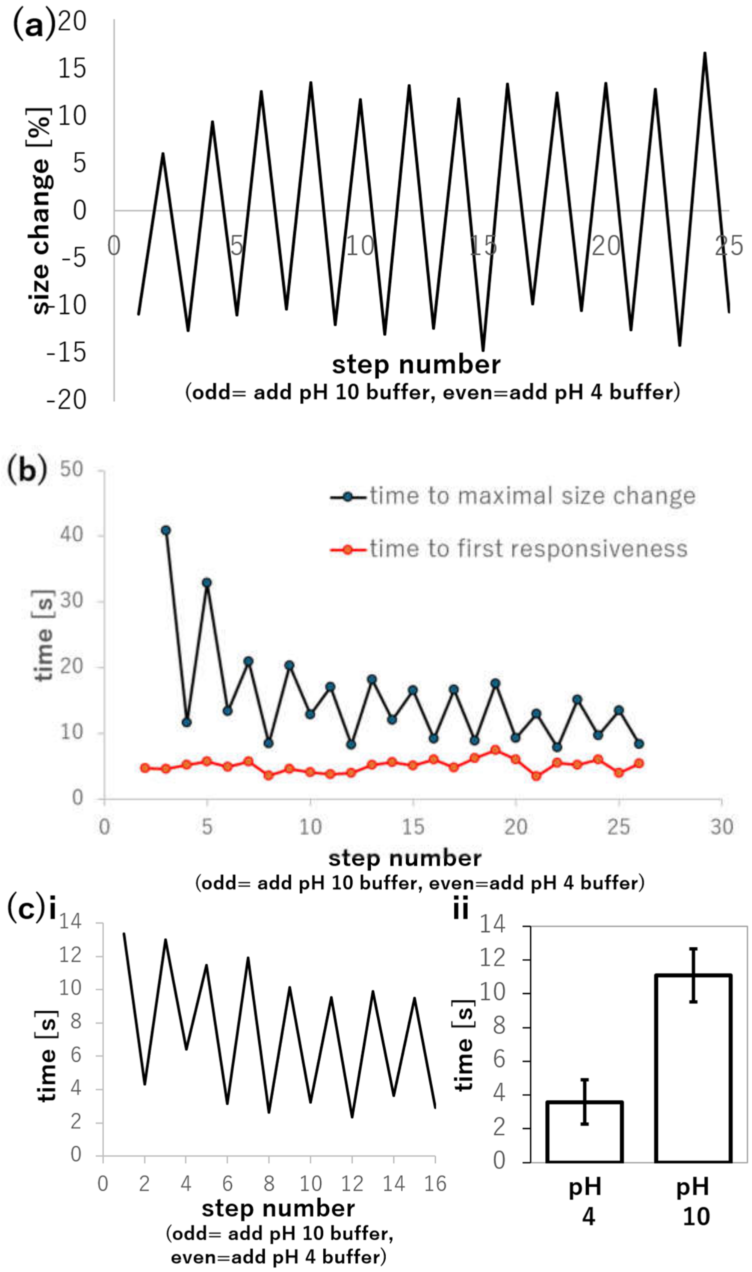

Figure S2: Response time. a-b) Data from table S5 is shown for a) size change and b) response time. a) In the first couple of steps size change in pH 10 buffer was not stable yet. b) The black line shows time to maximal size change. The red line shows time to initial response. c-i) Considering the first 10 steps as washing steps, the remaining 15 steps are shown. ii) The averages of these 15 steps are shown. Average response times are 3.6±1.3 s for a pH 10 to pH 4 change and 11.1±1.6 s for a pH 4 to pH 10 change.

4.3 anchorage

Table S5: Images of meshes before and after use.

<table>
<tr><td colspan="2">Extreme rough handling, several times air – buffer transitions, including blowing the channels content out with a hand blower. Mesh stayed intact, but it attached to the channel bottom due to air exposure.</td></tr>
<tr><td>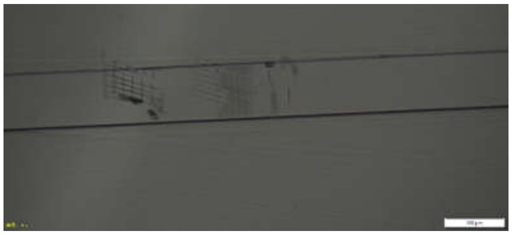</td><td>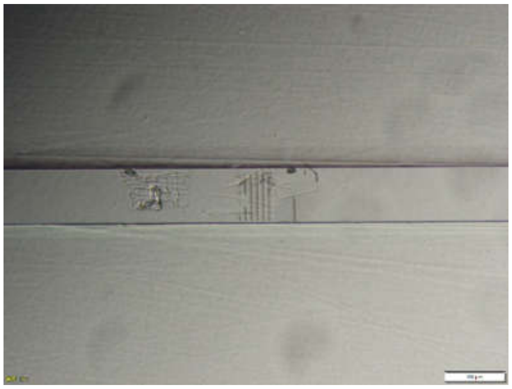</td></tr>
<tr><td colspan="2">Meshes spanning 500μm-wide channel. One mesh detached fully, one mesh detached half.</td></tr>
<tr><td>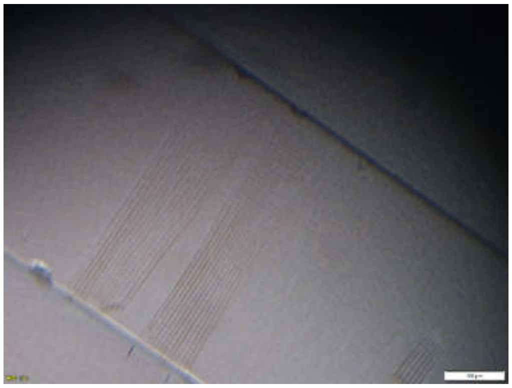</td><td>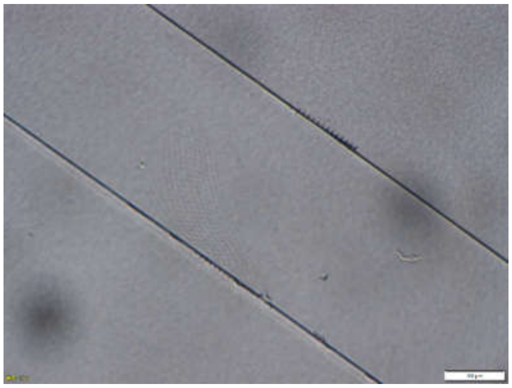</td></tr>
</table>

Horizontal mesh with well-stitched anchorage first and final images after 25 steps.

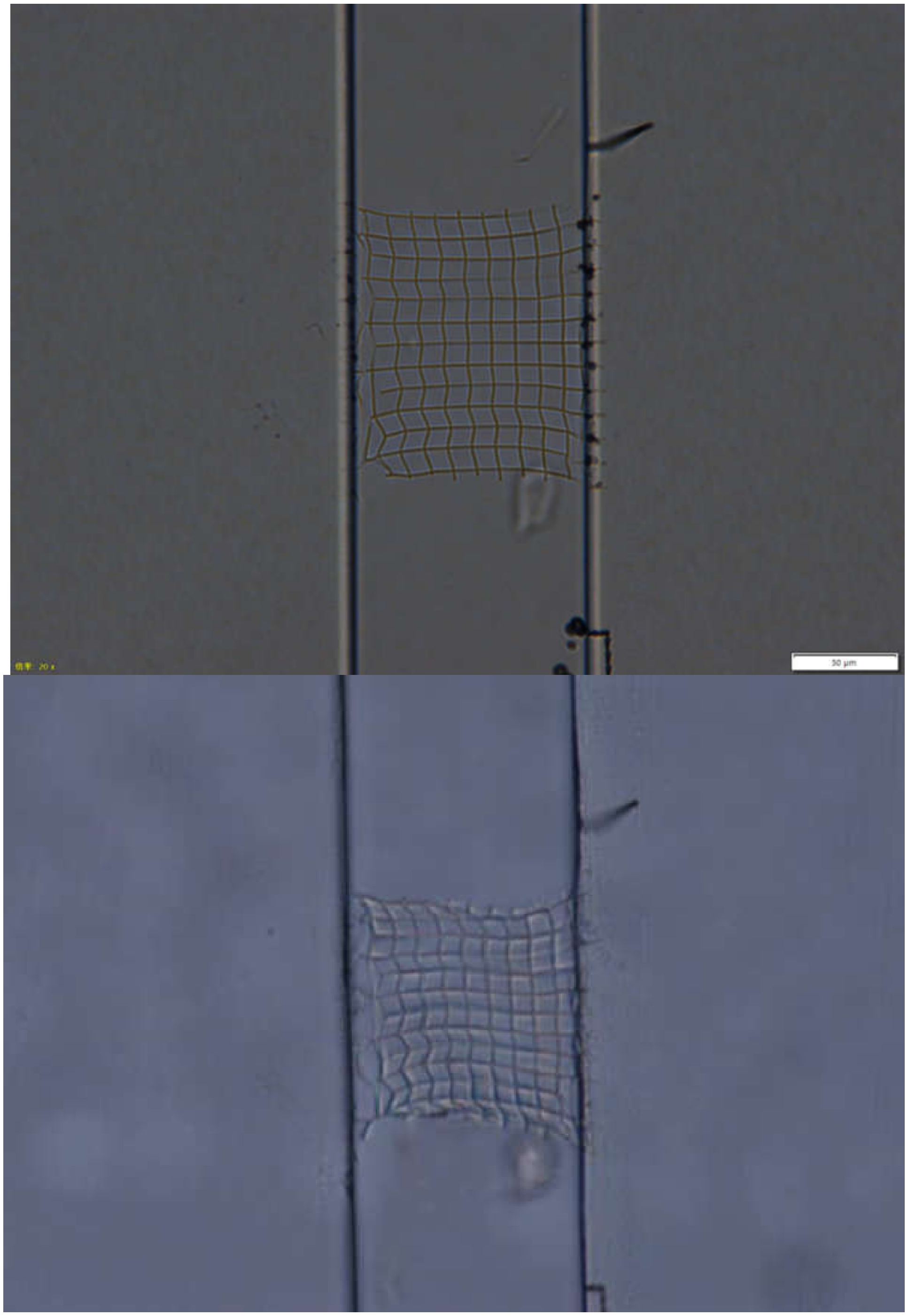

Supplement: Supplementary file 1 [file nanomaterials-15-00104-s001.zip › nanomaterials-3368517-supplementary.pdf]
